# Chronic Malaria Revealed by a New Fluorescence Pattern on the Antinuclear Autoantibodies Test

**DOI:** 10.1371/journal.pone.0088548

**Published:** 2014-02-13

**Authors:** Benjamin Hommel, Jean-Luc Charuel, Stéphane Jaureguiberry, Laurent Arnaud, Regis Courtin, Petra Kassab, Virginie Prendki, Luc Paris, Pascale Ghillani-Dalbin, Marc Thellier, Eric Caumes, Zahir Amoura, Dominique Mazier, Lucile Musset, Pierre Buffet, Makoto Miyara

**Affiliations:** 1 Immunology Department – Immune Chemistry and Autoimmunity Laboratory, Groupe Hospitalier Pitié-Salpêtrière, AP-HP, Paris, France; 2 Parasitology-Mycology Laboratory, Assistance Publique Hôpitaux de Paris, Groupe Hospitalier Pitié-Salpêtrière, Paris, France; 3 Tropical Infectious Diseases Department, Groupe Hospitalier Pitié-Salpêtrière, Assistance Publique-Hôpitaux de Paris, Université Pierre et Marie Curie, Paris, France; 4 INSERM (National Institute of Health and Medical Research) UMR-S 945, Paris, France; 5 Internal Medicine Department, French National Reference Center for SLE and Antiphospholipid Syndrome AP-HP Hôpital Pitié-Salpêtrière, Paris, France; 6 Department of Internal Medicine and Septic Orthopaedics, Hôpital Croix Saint-Simon, Paris, France; Mahidol-Oxford Tropical Medicine Research Unit, Thailand

## Abstract

**Background:**

Several clinical forms of malaria such as chronic carriage, gestational malaria or hyper-reactive malarial splenomegaly may follow a cryptic evolution with afebrile chronic fatigue sometimes accompanied by anemia and/or splenomegaly. Conventional parasitological tests are often negative or not performed, and severe complications may occur. Extensive explorations of these conditions often include the search for antinuclear autoantibodies (ANA).

**Methods:**

We analysed fluorescence patterns in the ANA test in patients with either chronic cryptic or acute symptomatic malaria, then conducted a one-year prospective study at a single hospital on all available sera drawn for ANA detections. We then identified autoantibodies differentially expressed in malaria patients and in controls using human protein microarray.

**Results:**

We uncovered and defined a new, malaria-related, nucleo-cytoplasmic ANA pattern displaying the specific association of a nuclear speckled pattern with diffuse cytoplasmic perinuclearly-enhanced fluorescence. In the one-year prospective analysis, 79% of sera displaying this new nucleo-cytoplasmic fluorescence were from patients with malaria. This specific pattern, not seen in other parasitic diseases, allowed a timely reorientation of the diagnosis toward malaria. To assess if the autoantibody immune response was due to autoreactivity or molecular mimicry we isolated 42 autoantigens, targets of malarial autoantibodies. BLAST analysis indicated that 23 of recognized autoantigens were homologous to plasmodial proteins suggesting autoimmune responses directly driven by the plasmodial infection.

**Conclusion:**

In patients with malaria in whom parasitological tests have not been performed recognition of this new, malaria-related fluorescence pattern on the ANA test is highly suggestive of the diagnosis and triggers immediate, easy confirmation and adapted therapy.

## Introduction

More than 2.2 billion people are exposed every year to *Plasmodium falciparum* infection, among whom 500 million experience clinical attacks with potential evolution toward severe, lethal disease. The World Health Organization has established that malaria caused 660 000 deaths per year, although this is likely an underestimate [1].

*P. falciparum* infection in humans induces a wide continuum of manifestations from acute severe attacks to completely asymptomatic status through subacute/chronic cryptic forms. A high proportion of people living in malaria-endemic countries have circulating malaria parasites without being frankly symptomatic [2], [3]. However, not all of them will have a benign evolution. Indeed *P. falciparum* causes several chronic entities that can lead to severe complications or even cause death. In non-endemic countries, 2.3 to 4.6% of patients with *P. falciparum* infection had been carrying the parasite for more than two months after leaving the endemic area [4], [5]. These late onset malaria due to *P. falciparum,* occurs in new immigrants from endemic areas and in pregnant women [5]. This occurs more frequently in patients of African origin (who are probably immune) and in pregnant women [5]. Malaria in pregnancy can cause severe complication to the mother and the fetus [6]. When women with prior immunity to malaria are infected with *P. falciparum* (gestational malaria) the main manifestation is low birth weight in the newborn, an important risk factor of infant mortality. Subacute, initially mild malaria attacks can occur in patients taking suboptimal antimalarial prophylaxis, for example with an antimalarial agent to which most parasites are resistant [7]. A cryptic form of malaria associated with chronic paucisymptomatic carriage of *P. falciparum* is Hyperreactive malarial splenomegaly (HMS). Typical HMS associates massive splenomegaly, high serum concentrations of polyclonal IgM and high titers of antimalarial antibody [8], [9]. Patients with HMS often carry cryoglobulins [10], may suffer from acute episodes of antibody-mediated hemolytic anemia [11], and develop acute malaria attacks after splenectomy [9], [12]. Splenic lymphoma emerges after years of untreated evolution [13] and infectious complications occur, likely because of impaired splenic function. In early descriptions of HMS in New Guinea mortality rates as high as 36% have been reported during a two-year follow up of 99 patient [14]. Most patients with these cryptic forms of *P. falciparum* infection carry very low parasite loads, often undetectable by conventional parasitological methods [9], [12], [15] and therefore come to the hospital with prolonged complaints such as low-grade fever, fatigue, anemia, splenomegaly not immediately suggestive of malaria. These situations are particularly misleading because the last visit to an endemic area often occurred more than three months, sometimes several years before the onset of symptoms [5], justifying extensive laboratory explorations often including the search for antinuclear autoantibodies (ANA). The presence of ANA with speckled pattern [16], [17], [18], [19] as well as cytoplasmic fluorescence on anti-neutrophils cytoplasmic antibodies (ANCA) testings [20], [21] have been described in malaria but are not specific of this diagnosis. Here, we report an original fluorescence pattern on HEp2000™-cells defined as nuclear speckled with diffuse cytoplasmic pattern and perinuclear enhancement that is related to *P. falciparum* infection. Its recognition recently allowed the diagnosis to malaria in several patients at our center. We also show that polyclonal autoantibodies of IgG isotype are responsible for this pattern recognizing auto-antigens with high homology with plasmodium proteins.

## Materials and Methods

### Patients and Biological Materials

Sera from fourteen patients (nine Caucasian expatriates and five immigrants from Africa) with HMS followed up in infectious disease department (2005–2011) and from 22 patients with non HMS *P. falciparum* malaria (21 with acute malaria and one with gestational malaria) were collected at −20°C in the national reference center from malaria. Serum IgGs were isolated by G-protein affinity chromatography (NAb Spin kits, Pierce).

Control sera with elevated IgM levels were collected from ten Waldenström disease patients. Control sera from parasitic diseases were collected at −20°C in the parasitology-mycology laboratory serum bank (2004–2011). These were obtained from patient who had positive serology for toxocariasis (50 patients), filariasis (8 patients), schistosomiasis (40 patients), visceral leishmaniasis (10 patients) and Chagas disease (44 patients). Samples drawing was carried out according to local ethics committee regulations and ethical approval was obtained from the “Comité de protection des personnes-Ile de France-VI” at the Pitié-Salpêtrière Hospital. No consent was needed from any patients involved in this study (bioethics law n° 2011–814, july 2011 article 17). Patients sera were routinely analyzed for diagnosis procedures. Subsequent Western blot and and protein array experiments were performed on selected anonymized samples.

10,400 consecutive serum samples from patients referred to Pitié-Salpêtrière Hospital (Paris, France) for diagnostic procedures for systemic autoimmune diseases, drawn from January 2011 to January 2012 were processed in a non-blinded manner by at least two independent observers using a LEICA/DM-LB2 (400 magnification). ANA titer was determined by testing successive two-fold dilutions of the serum from 1/80 to 1/1280. Samples were classified as ANA positive if well-defined immunofluorescence patterns were identified at 1/160 dilution for nuclear and 1/80 for cytoplasmic fluorescence respectively.

### Indirect Immunofluorescence on HEp-2000™ Cells and Human Neutrophils for the Detection of ANA and ANCA

Serum samples from all participants were subjected to the ANA-test using commercial slides (Immunoconcept) and a PhD system immunoassay (Biorad). HEp-2000™ are HEp-2 cells with overexpressed Ro60 antigens. Serum samples were diluted in PBS buffer (H_3_PO_4_ 0.01 M, pH 7.4±0.2, Immunoconcept) and incubated for 25 minutes at room temperature in a moist chamber. After washing twice in PBS, cells were incubated with fluorescein isothiocyanate conjugated goat anti–human Ig G(Ig heavy and light chain, Immunoconcept) for another 25 minutes in the dark. After washing twice as before, slides were counter-stained with Evans blue and assembled with glycerol and coverslips. Some samples were also processed for the ANCA-test using commercial NOVA Lite® ANCA ethanol or formalin slides. 30 µL of 1/20 diluted sample were spotted on each well, then incubated for 25 minutes and washed with PBS buffer (Immunoconcept). Cells were subsequently incubated with fluorescein isothiocyanate conjugated goat anti–human Ig and incubated for 25 minutes in the dark. After washing twice as before, slides were counter-stained with Evans blue and assembled with glycerol and coverslips. ANCA-test and ANA-test require the agreement between the blinded observation of both observers to minimize the influence of subjectivity.

### *P. falciparum* Specific Indirect Immunofluorescence Assay

Positive *P. falciparum* thin smear slides (>1% parasitaemia) frozen at −80° were immersed in acetone and dried. Patient sera were diluted in PBS buffer (pH = 7.2, NaCl 6.80 g (Prolabo) Na_2_ HPO_4_ 1.48 g (Prolabo), KH_2_PO_4_ 0.43 g (Prolabo)) from 1/200 (cut-off for positive samples) to subsequent increasing dilution. 40 µL of samples were spotted on each slides well, incubated for 30 minutes in a humid atmosphere and then washed in PBS buffer. 40 µL of fluorescein isothyocyanate-conjugated anti-human IgG (1 ml of PBS, 10 µL of anti-globulin (MALARIA: IgG Anti-NORDIC (Gahu/Ig/FITC)), 50 µL of Evans Blue) were subsequently added and incubated for 30 minutes in a humid atmosphere and washed with PBS buffer.

### Polymerase Chain Reaction

The *Plasmodium* species-specific real-time PCR is a multiplex PCR for the determination of the four different *Plasmodium* species. It was realized as described previously [22].

### Western Blotting on HEp-2 Cell Lysates

HEp-2 cells were cultured in Dulbecco’s modified eagle medium (DMEM) supplemented with 2% L-glutamine, 5% bicarbonate pH 7.5, 2.4% HEPES, 10% fetal calf serum, 1% penicillin streptomycin amphotericin B. Cells were maintained at 37°C in 5% CO_2_ humidified atmosphere and were fed every 3 days with fresh medium. First, on ice, cells were rinsed for residual medium with a solution 0.01M sodium phosphate, 0.15M NaCl pH 7.2. Thus, cells lysis was obtained using detergent buffer (0.025M Tris, 0.15M NaCl, 0.001M EDTA, 1% NP-40, 5% glycérol; pH 7.4) for 5 minutes at 4°C. To avoid protease reaction an inhibitor cocktail (Sigma-Aldrich) was added. The whole lysate was ultracentrifuged at 13 000 g for 10 minutes and the supernatant was immediately frozen at −40°C. Proteins concentration was determined at 595 nm using the Bio-Rad protein assay reagent.

IgGs were isolated from the sera by affinity chromatography. Briefly, serum samples were treated by micro columns coated with G-protein using NAb Spin kits, 0.2 mL for Antibody purification (Pierce).

For western blotting whole cell extract were heated for 4 minutes at 100°C and 100 µg were loaded onto 12% SDS-polyacrylamide gel (Biorad). The gel was subjected for 2 h30 electrophoresis at 100 v and transferred by electroblotting to polyvinylidene difluoride (PVDF, Millipore) membranes overnight at 30 v. Membranes were blocked 1 hour at room temperature with blocking buffer (PBS with 0.5% tween 20 and 5% skim milk powder) and incubated for 1 h with purified IgG antibody from patient sera (dilution: 1/100). Thus, blots were incubated for 1 h at room temperature with a secondary horseradish peroxydase conjugated anti human antibody (goat polyclonal anti human, Mpbiomedicals). Blots were scanned after using the enhanced chemiluminescence method (Biorad) on an Ettan Dige imager (GE healthcare). The measurement of the number of bands was done by independant observers, each group was blinded before counting.

### Human Protein Microarray

Targets of the IgGs were isolated from 3 patients with HMS and from pooled sera of 5 patients with acute malaria. There were compared with the targets of IgGs from 5 healthy donors using a protein array and following the protocol recommended by the manufacturer (ProtoArray® Application Guide- Invitrogen™ : Immune Response Biomarker Profiling). Microarrays were readen by Invitrogen™ and raw data were sent for further exploitation. Identification of differentially expressed autoantibodies targets between malaria patients and controls was performed using FlexArray version 1.6.1 (McGill University & Génome Québec Innovation Centre, Montreal, Canada). The raw data were adjusted for background signal and normalized across all Protoarray chips using the quantile method. Differentially expressed autoantibodies were identified using the Statistical Analysis of Microarray (SAM) algorithm [**23**] with a false discovery rate correction performed using Benjamini-Hochberg method. Each of the 9400 protein targets were plotted on a volcano plot. Targets with a fold change superior to 8 with a statistical significance inferior to 0.01 that we considered as highly specific for malaria are shown in the chart. For each target, the cellular localization of the protein is indicated.

### Statistical Analysis

Statistical analyses were made using Student, Khi2, Mann-Whitney, logistic regression tests and D’Agostino & Pearson omnibus to test normality. Results were considered statistically significant at p<0.05.

## Results

### Index Case: a Man Presenting with Chronic Splenomegaly and Autoantibodies with a Distinct Nucleo-cytoplasmic Pattern Detected by Indirect Immunofluorescence on HEp-2000™ Cells

A 42 year-old man, born in Mali, living in France for the last 10 years, requiring chronic dialysis for end stage renal disease secondary to hypertensive nephrosclerosis, was referred to the internal medicine department for the occurrence of arthralgia, thrombocytopaenia, splenomegaly and the presence of autoantibodies in biological testings.

**Figure 1:**
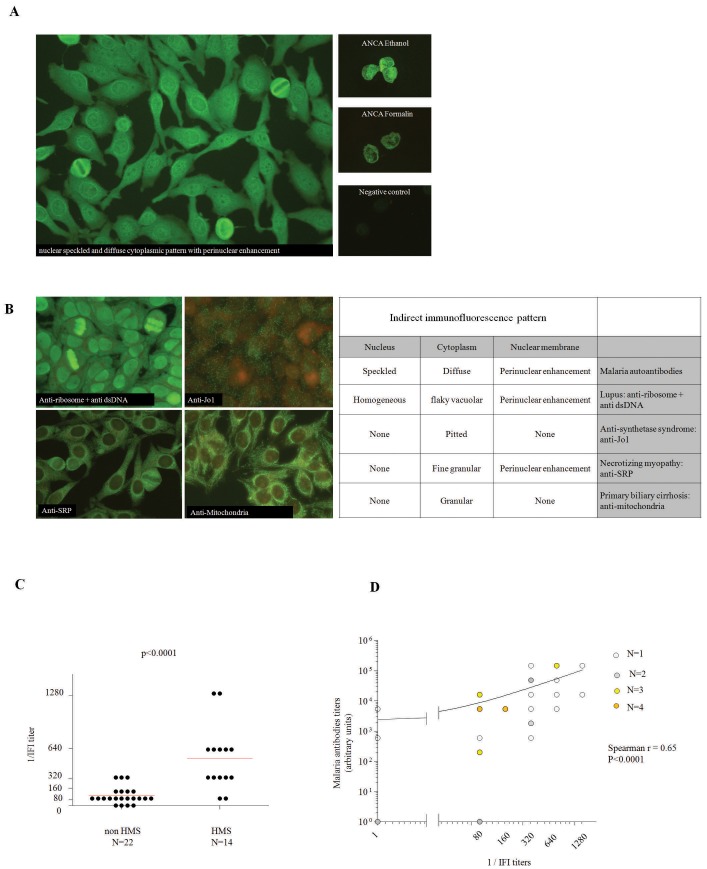
Distinct immunofluorescence pattern on HEp-2000™ cells detected in the serum of patients with malaria. **A** Nuclear speckled and cytoplasmic diffuse pattern with perinuclear enhancement detected by indirect immunofluoresence on HEp-2000™ cells (left) and on human neutrophils fixed with ethanol (top right) with the serum of a patient with HMS. Cytoplasmic diffuse pattern with perinuclear enhancement on neutrophils fixed with formalin. The nuclear speckled pattern is faded as observed with usual antinuclear antibodies (right middle). Negative control for ANCA testing (right bottom). **B** Common immunofluorescence patterns with cytoplasmic fluorescence that are diagnostic for SLE (anti-ribosome+anti-DNA), anti-synthetase syndrome (Anti-Jo1), necrotizing myopathies (anti-SRP) and primary biliary cirrhosis (anti-mitochondria) (left) compared with the distinct immunofluorescence pattern observed with malaria autoantibodies (right). **C** titers of autoantibodies with the distinct nucleo-cytoplasmasmic pattern in patients with HMS (n = 14) or non-HMS malaria (n = 22). Comparison performed using a U Mann-Whitney non parametric test. **D** Correlation of the titers of anti-malarial antibodies with the titers of autoantibodies with the distinct nucleo-cytoplasmic pattern. Correlation assessed using a Spearman non parametric test.

Detection of ANA revealed an atypical diffuse cytoplasmic with perinuclear enhancement and nuclear speckled fluorescence at a 1/640 titer on HEp-2000™ cells, while detection of ANCA revealed an atypical perinuclear/cytoplasmic pattern without anti-PR3 or anti-MPO specificities (**FIGURE 1**).

Because of the presence of splenomegaly and thrombocytopaenia and the patient’s country origin, thin and thick blood film were performed and returned very low positive (parasitaemia<0.01%). High levels of serum IgM were observed as well as high titer of antibodies against *P. falciparum* antigens (**TABLE 1**).

**Table 1:** Index case: Clinical and biological characteristics.

**Age**	42		
**Gender**	M		
**Symptoms**	Splenomegaly		
	Arthralgia		
**Hematocrit (%)**	37	IgG (g/L)	21.7
**Hemboglobin (g/dl)**	12	IgA (g/L)	1.4
**White-cell count (G/L)**	3.41	IgM (g/L)	18.5
**Differential count**			
**Neutrophils (G/L)**	1.8	Cryoglobulinaemia	polyclonal
**Lymphocytes (G/L)**	1.3	Rheumatoid factor	39 UI/ml
**Monocytes (G/L)**	0.13		
**Eosinophils (G/L)**	0.1	Antinuclear antibodies	
**Basophils (G/L)**	0	antiSSA	negative
**Platelet count (G/L)**	100	antiSSB	negative
**Mean corpuscular volume fl**	81.7	Anti-Sm	negative
**Sodium (mmol/L)**	137	Anti-RNP	negative
**Potassium (mmol/L)**	5.4	Anti-SCl70	negative
**Chloride (mmol/L)**	106	Anti-centromere	negative
**Carbon dioxide (mmol/L)**	20	Anti-PCNA	negative
**Urea nitrogen (mmol/L)**	20.9L	Anti-DNA	negative
**Creatinine (µmol/L)**	673		
**Bilirubin (µmol/L)**	7	ANCA	cytoplasmic
**Phosphorus (mmol/L)**	1.03	anti-PR3	negative
**Calcium (mmol/L)**	2.19	anti-MPO	negative
**Aspartate aminotransferase (U/I)**	22		
**Alanine aminotransferase (U/I)**	12	malarial serology titer	5400
**CRP (mg/L)**	<4	thin/thick blood smear	*P. falciparum* parasitemia<0.01%

The patient met the diagnostic criteria for HMS and short term malarial therapy was introduced (association of atovaquone/proguanil) during three days which led to a rapid recovery. The patient has been followed up for two years and a rapid decrease in the concentration of serum IgM returning to normal values within months was observed while the titers of autoantibodies on ANA testings remained at high levels with similar fluorescence patterns.

### Presence of a Distinct Nucleo-cytoplasmic Pattern on ANA Testings in Patients with HMS

The distinct nucleo-cytoplasmic pattern observed with the serum of the index case described above, is different from the cytoplasmic and nuclear patterns that are usually observed in some connective tissue diseases (anti-synthetase syndrome with anti-Jo1, necrotizing myositis with anti-SRP, systemic lupus erythematosus (SLE) with anti-RiboP autoantibodies or primary biliary cirrhosis with anti-mitochondria antibodies) shown in **FIGURE 1**.

We first hypothesized that this distinct pattern is observed in HMS. We thus retrospectively analysed the sera of 14 patients with defined HMS and 22 patients with non-HMS malaria, i.e. recent acute malaria or reported history of acute malaria (**TABLE 2**). Because all patients with HMS are characterized by high levels of serum IgM, we also selected ten sera drawn from patients with Waldenström macroglobulinaemia to verify that the pattern was not artefactually due to the presence of high IgM levels.

**Table 2:** Patients with malaria: Clinical and biological characteristics.

	non HMS (n = 14)	HMS (n = 22)	bivariate logistic regression (p value)
**Age mean ± SD**	42.8±12.7	66.8±15.7	
**Gender (M/F)**	17/5	10/4	
**Splenomegaly (+/−/ND)**	1/9/12	12/1/1	
**Hepatomegaly (+/−/ND)**	0/8/14	7/2/5	
**IgG mean ± SD (g/L)**	16.1±3.7	20.7±4.9	0.014
**IgA mean ± SD (g/L)**	2.7±1.1	2.5±1.7	0.69
**IgM mean ± SD (g/L)**	2.7±1.7	9.2±5.0	0.001
**Positive Malarial serology (%)**	81.8	100	
**ANA mean fluorescence titer ± SD**	400±280	822±429	0.015
**Distinct nucleo-cytoplasmic pattern mean** **fluorescence titer ± SD**	123±422	582±106	0.011
**Positive cytoplasmic ANCA (%) (+/−/ND)**	22.2 (4/18/4)	42.8 (6/14/0)	

While we did not observe the distinct nucleo-cytoplasmic pattern with any of the sera of patients with Waldenstrom macroglobulinaemia (data not shown), we observed this pattern with the sera of all patients with HMS (n = 14, mean titer: 537±SD373). This pattern was observed at lower titers with the sera of 18/22 patients (81.2%, mean titer: 112±SD98, p<0.0001) with non-HMS malaria (**FIGURE 1C**). Of note, an atypical pattern could also be detected on ANCA immunofluorescence assay as observed with the serum of the index case patient **(Figure S1)** There were 10 patients positive for the atypical pattern over the 34 tested (all patients could not be tested because of the lack of serum). Finally, the titers of the nucleo-cytoplasmic pattern on Hep-2000™ cells were significantly correlated with the titers of antibodies against *P. falciparum* antigens (**FIGURE 1D**).

Because parasitic diseases are known to induce autoantibodies [24], [25], [26], [27] selected sera from patients with either schistosomiasis, toxocarosis, chagas disease, filariasis or leishmaniasis were tested.

The distinct nucleo-cytoplasmic pattern was not observed with any of these sera **(table S1**).

These findings indicate that a distinct nucleo-cytoplasmic pattern is observed in most malaria patients and especially at a high titer in HMS patients. We thus thereafter refer to this pattern as malaria-related pattern.

### Four Patients Diagnosed by the Malaria-related Pattern in a One Year 10,400 ANA Test Prospective Study

**Figure 2:**
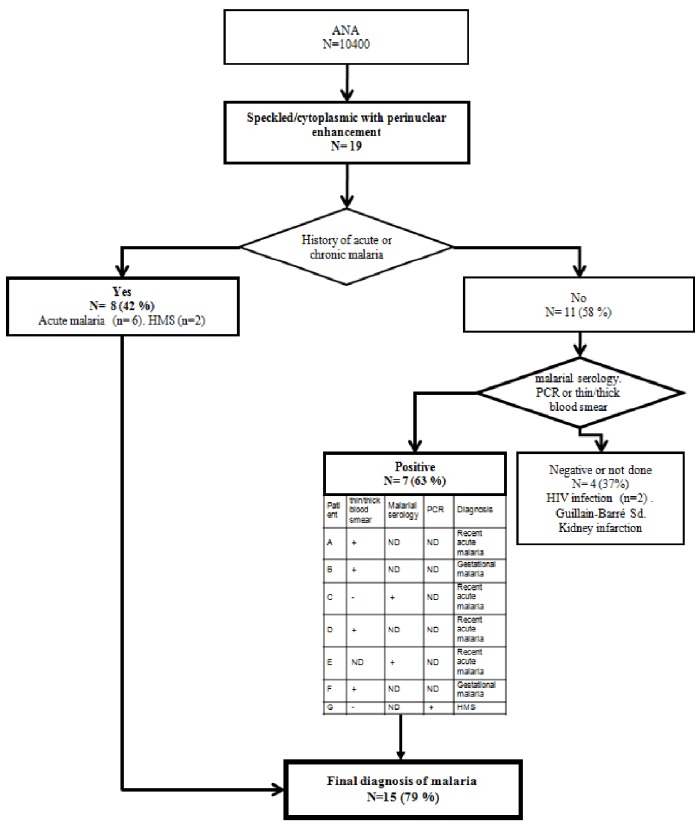
Nuclear speckled and cytoplasmic diffuse with perinuclear enhancement pattern is diagnostic for smoldering malaria. Occurrence or past history of malaria in patients with the distinct nucleo-cytoplasmic pattern was prospectively studied. The pattern was observed in 19 independent sera out of 10 400 ANA tests performed over a 12 month-period. For each patient, the occurrence of malaria was first searched in the medical clinical record file. If history of malaria was absent, serology, PCR or thin/thick blood smear were performed. Diagnosis for each case is indicated. (ND = not done).

To assess the diagnostic value of the distinct nucleo-cytoplasmic pattern for malaria, we prospectively studied 10,400 consecutive ANA tests performed for a year in the laboratory, among which 19 sera displayed malaria-related pattern i.e 1.6 sera per month. We designed the study as a blind test for malaria diagnosis since the examiners for ANA test were not aware of the clinical history of the patients. When a malaria-related pattern was recognized, medical records of the patients were reviewed to detect a clinical history of recent or chronic malaria and referral physicians were asked to consider malaria as a possible diagnosis and suggested to have diagnostic tests for malaria diagnosis performed. Among the 19 patients with the distinct nucleo-cytoplasmic pattern, eight patients had an already known history of malaria (42%) with recent acute malaria (n = 6) or chronic malaria (n = 2). Among the 11 patients with unknown previous occurence of malaria, seven patients had a test positive for malaria: four with positive thin and think smear, two with positive malaria serology and one with a positive polymerase chain reaction (PCR) for *P. falciparum.* Three of the latter patients had ongoing acute malaria first diagnosed by the clinicians while the ANA result specifically prompted the unexpected diagnosis of four active infections not previously reported in the clinical files or disclosed by the patient (one acute malaria and three chronic forms including two gestational malaria) (**FIGURE 2**). All patients underwent specific treatment for malaria.

Altogether, these findings indicate that the detection of malarial autoantibodies with a distinct nucleo-cytoplasmic pattern may be useful for the diagnosis of asymptomatic smoldering malaria.

Among the 11 patients whith unknown occurrence of malaria 4 had the nucleo-cytoplasmic pattern and were not found to correspond to malaria. While one patient had a kidney infarction of unknown cause, the three others had immune diseases (2 HIV and a Guillain Barré syndrome).

### Polyclonal Autoantibodies of IgG Isotype Responsible for Malaria Related Pattern: Identification of Autoantigens Targets Highly Homologous with Plasmodium Proteins

We investigated possible autoantibodies targets that are responsible for the nucleo-cytoplasmic pattern in *P. falciparum* infected patients.

First, we verified that autoantibodies were of IgG isotype. This was as predicted because secondary fluorescent antibodies in the ANA assays recognize human IgG. Indeed, the nucleo-cytoplasmic patterns on HEp-2000™ cells were similar on ANA tests with purified IgG from malaria patients sera to the pattern observed with the whole sera. This further indicates that malaria autoantibodies are of IgG isotype (**FIGURE S2**).

**Figure 3:**
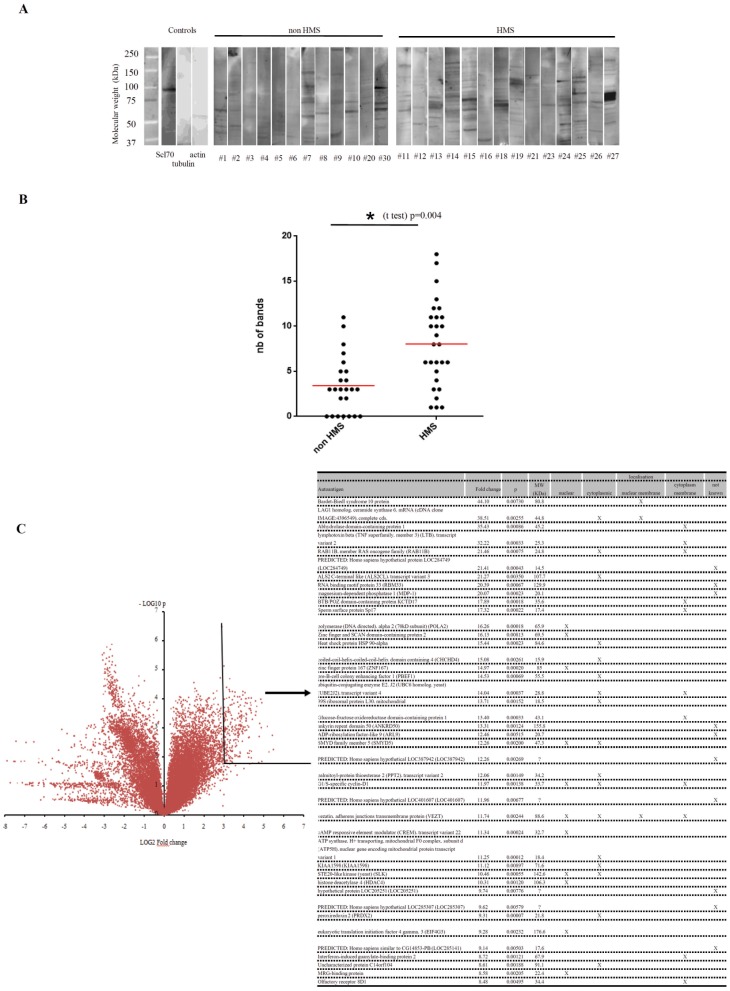
Polyclonal autoantibodies of IgG isotype are responsible for the malaria related pattern. **A** Detection by Western blotting of autoantibodies in the serum of malaria patients recognizing HEp-2 cells proteins. Each lane corresponds to an individual experiment with the serum obtained from indicated patient. Positive control are shown with anti-actin and anti-tubulin monoclonal antibodies and anti-Scl70 positive serum obtained from a patient with systemic sclerosis. **B** Comparison of the number of bands observed on Western blotting gels in non-HMS (n = 12) and HMS (n = 14) malaria. Comparison made using a non-parametric U Mann-Whitney test. **C** Determination of the targets of malarial autoantibodies. Targets of the IgGs isolated from three patients with HMS and from pooled sera of five patients with acute malaria were compared with the targets of IgGs from five healthy donors using a protein array (four experiments for malaria and five for healthy donors). Each of the 9 400 protein targets were plotted on a volcano plot. Targets with a fold change superior to eight with a statistical significance inferior to 0.01 that we considered as highly specific for malaria are shown in the chart. For each target, the cellular localization of the protein is indicated.

To determine whether the autoimmune IgG responses **are** mono or poly-specific (i.e. recognizing only one or several autoantigens), we performed a Western blot analysis of whole cell protein extracts of HEp-2 cells with isolated IgGs (**FIGURE 3A**). We first noticed that none of the IgG samples, extracted either from HMS or non-HMS malaria patients, recognized a single target. Furthermore, the number of bands was superior with the sera of HMS patients compared to non-HMS malaria patients (**FIGURE 3B**). Thus, the autoimmune IgG response in malaria is polyclonal and polyspecific and more intense in HMS.

To identify the autoantigens recognized by malaria IgG autoantibodies, we performed a protein array analysis on individual samples of isolated IgG from three HMS patients (#14, #15 and #26), and pooled samples of isolated IgGs from five patients with acute malaria (#1–5). The intensity signals comparison obtained with malaria IgG with the control IgG (healthy donors’ sera) enabled the identification of 42 autoantigens out of the 9 400 potential targets with a fold increase >8 and a strong statistical significance (p<0.01). Among these targets, 11 proteins were of intranuclear, three of nuclear membrane and 17 of cytoplasmic localization (**FIGURE 3C**). In silico alignment analysis of recognized protein with plasmodial proteins indicated that 23 recognized autoantigens were homologous with proteins of *P. falciparum* (**Table S2**).

## Discussion

Although the symptoms of acute malaria are generally recognized by trained physicians, malaria may be overlooked when the patients have visited or left endemic areas months or years before [28]. The risk of missing the diagnosis is even higher when malaria adopts a smoldering form like chronic carriage, gestational malaria or HMS. In these situations the clinical presentation is subacute or chronic, and conventional parasitological tests may be negative. These patients are therefore sometimes investigated for several days or weeks in internal medicine or other settings for fatigue, splenomegaly, anemia, cytopenia, hemophagocytic syndrome [29], or can be mistakenly splenectomized with a suspicion of splenic lymphoma [9], [12]. We show here that a distinct nucleo-cytoplasmic pattern on indirect immunofluorescence i.e. cytoplasmic diffuse, nuclear speckled with perinuclear enhancement fluorescence while searching for ANA can efficiently reorient the diagnosis toward malaria. Rapid confirmation of the diagnosis is then with antimalarial serology or amplification of *P. falciparum* nucleic acids. Our prospective analysis in 10,400 patients confirms that even a patient with unknown history of malaria is likely to have a smoldering form of malaria if his serum displays the nucleo-cytoplasmic malaria-related pattern when searching for ANA. Indeed, 15 of 19 sera (79%) displaying the malaria-related pattern were from patients with either past or active malaria. Although some patients with the distinct nucleo-cytoplasmic pattern had a previously cured infection (six cured from acute malaria and two with previous HMS [42%]), we uncovered four active infections not previously reported in the clinical files or disclosed by the patient. Even more importantly, the pattern revealed among the latter unexpected diagnosis of ongoing gestational malaria in two patients and active HMS in one. Preliminary results in three patients suggest that *P. malariae*, and *P. ovale* may induce the same nucleo-cytoplasmic malaria-related pattern in the ANA test (data not shown).

Detection of ANA is routinely performed for the diagnosis of systemic autoimmune diseases such as SLE [30]. While the detection of autoantibodies recognizing components of the nucleus have been extensively tested for more than 50 years, autoantibodies recognizing components of the cytoplasm have not drawn much attention until the identification in the late 80′s of anti-ribosome autoantibodies in SLE and anti-Jo1 autoantibodies in inflammatory myositis [31]. Other distinct cytoplasmic patterns have been also associated with autoantibodies found in other autoimmune myositis (anti-SRP and other anti-tRNA synthetase antibodies) [32], [33] and in primary biliary cirrhosis (anti-mitochondria autoantibodies) [34]. Thus both nuclear and cytoplasmic patterns can orient the diagnosis toward autoimmune diseases. Whereas presence of ANA has been reported in malaria since the 70′s [17] this is the first report of a distinct fluorescence pattern on Hep-2000™ cells that can be used to orient diagnosis toward smoldering malaria.

Titers of malarial autoantibodies by immunofluorescence, and the number of bands on HEp-2 cell Western blot analysis were higher in chronic malaria infection compared to acute attacks. The intensity of the autoimmune response may therefore be related to the prolonged persistence of the parasite. To ensure that the fluorescence pattern was related to the immune response to *P. falciparum* antigens, we identified targets of autoantibodies from eight patients using chips displaying 9,400 human proteins. We focussed the analysis on 42 autoantigens likely involved in the observed malaria-related pattern because of their high fluorescence scores and their nuclear, perinuclear or cytoplasmic localizations. BLAST analysis indicated that 23 of these 42 (54.7%) autoantigens were homologous to plasmodial proteins. This suggests that autoimmune responses could be triggered by a molecular mimicry between parasite and human proteins. Alternatively, a broad intense non-specific polyclonal stimulation, may lead to the production of antibodies truly directed against self-targets. Molecular mimicry and non-specific stimulation of anti-self B clones are not mutually exclusive mechanisms. Both mechanisms may explain the higher titers of malaria-related autoantibodies in malarial chronic diseases compared to acute diseases. Whether particular repertoires of malaria autoantibodies are correlated to the occurrence of different forms of malaria has yet to be determined.

Taken together, our findings indicate that a distinct nucleo-cytoplasmic pattern on ANA tests is diagnostic for *P. falciparum* infection in patients being explored in internal medicine or other settings such as obstetrics [35]. The suspicion of malaria triggered by the discovery of malaria related immunofluorescence pattern can be easily confirmed by conventional tests (thick and thin Giemsa-stained smears, rapid diagnostic test) or more accurately by PCR as parasite loads are often low in this particular context of smoldering malaria. We expect that reorientation of the diagnosis toward malaria will shorten management, spare expensive imaging and/or painful explorations leading to the cure of patients with a 3-day oral, effective non-toxic treatment [36]. Indeed, our findings could be verified after the completion of the prospective study since we could routinely uncover 12 new cases of malaria over an 18 month period (12 with positive *P. falciparum* serology and six with positive PCR for *P. falciparum*).

Not least, prompt diagnosis and treatment of malaria may prevent unjustified splenectomy or evolution towards splenic lymphoma in patients with HMS, may protect newborn from women with gestational malaria from low-birth weight and related complications [9].

## Supporting Information

Figure S1 **Presence of nuclear speckled and cytoplasmic diffuse pattern with perinuclear enhancement detected by indirect immunofluorescence on neutrophils in patients with malaria.** Presence of nuclear speckled and cytoplasmic diffuse pattern with perinuclear enhancement detected by indirect immunofluoresence on HEp-2000™ cells (left) and corresponding fluorescence on human neutrophils fixed with ethanol (right). Representative results of five patients are shown.
(TIF)

Figure S2 **Malaria autoantibodies are of IgG isotype.** Malaria related pattern on HEp-2 cells obtained with the whole serum (left) and corresponding isolated IgG (right). Representative results of five patients are shown.
(TIF)

Table S1 **ANA testings on HEp2000 cells of sera from patients with schistosomiasis, toxocarosis, leishmaniasis, filariasis or leishmaniasis.**
(DOCX)

Table S2 **Homology of autoantigens recognized by malalarial autoantibodies with plasmodial antigens using BLAST analysis.**
(DOCX)
