# Correction: Chronic Malaria Revealed by a New Fluorescence Pattern on the Antinuclear Autoantibodies Test

**DOI:** 10.1371/journal.pone.0092361

**Published:** 2014-03-07

**Authors:** The PLOS ONE Staff

Multiple affiliations for multiple authors are incorrect. The author apologizes for these errors. Please view the author list, with the correct affiliations indicated, below:

Benjamin Hommel^1,2^, Jean-Luc Charuel^1^, Stéphane Jaureguiberry^3,4^, Laurent Arnaud^4,5,6^, Regis Courtin^2^, Petra Kassab^1^, Virginie Prendki^7^, Luc Paris^2^, Pascale Ghillani-Dalbin^1^, Marc Thellier^2,4^, Eric Caumes^3,4,5^, Zahir Amoura^4,5,6^, Dominique Mazier^2,4,5^, Lucile Musset^1^, Pierre Buffet^2,4,5^, MakotoMiyara^1,4,6^

1 AP-HP, Hôpital Pitié-Salpêtrière, Immunology department- immune chemistry and autoimmunity laboratory, F-75013, Paris, France.

2 AP-HP, Hôpital Pitié-Salpêtrière, Parasitology-mycology laboratory, French National Reference Center for Malaria, F-75013, Paris, France.

3 AP-HP, Hôpital Pitié-Salpêtrière, Tropical Infectious Diseases departement, F-75013, Paris, France.

4 INSERM, U1135, CIMI, F-75013, Paris, France, Paris, France.

5 Sorbonne Universités, UPMC Univ Paris 06, CR7, Centre d'Immunologie et des Maladies Infectieuses (CIMI), F-75013, Paris, France

6 AP-HP, Hôpital Pitié-Salpêtrière, Internal Medicine Department, French national Reference center for SLE and antiphospholipid syndrome, F-75013, Paris, France.

7 Departement of Internal Medicine, Hôpitaux universitaires de Genève, Suisse.
