# Supplementary figures and images for: Chronic Malaria Revealed by a New Fluorescence Pattern on the Antinuclear Autoantibodies Test
Source: PLoS One. 2014 Feb 13;9(2):e88548. doi: 10.1371/journal.pone.0088548 (PMC3923801; doi:10.1371/journal.pone.0088548)

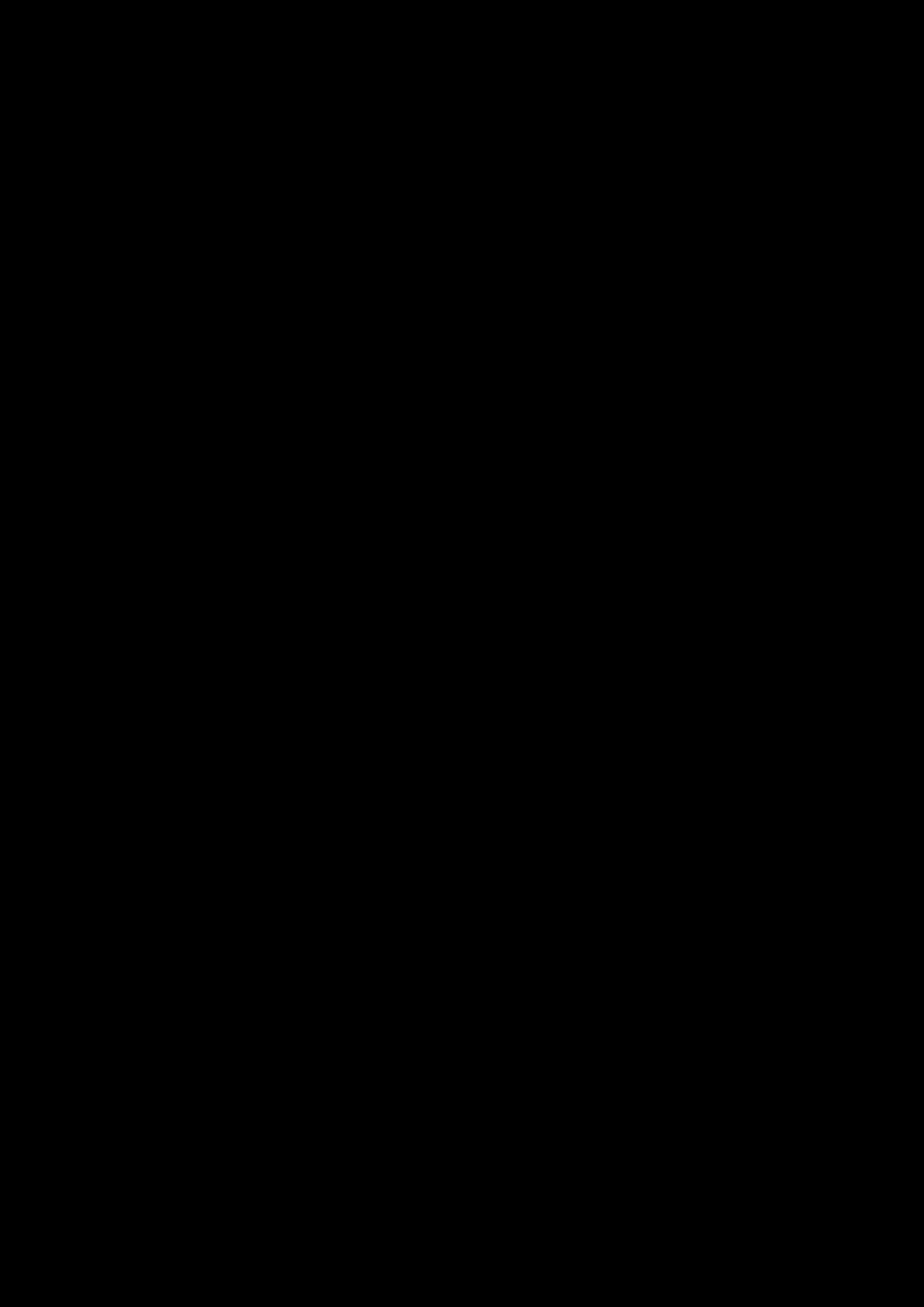

Supplement: Figure S1 — Presence of nuclear speckled and cytoplasmic diffuse pattern with perinuclear enhancement detected by indirect immunofluorescence on neutrophils in patients with malaria. Presence of nuclear speckled and cytoplasmic diffuse pattern with perinuclear enhancement detected by indirect immunofluoresence on HEp-2000™ cells (left) and corresponding fluorescence on human neutrophils fixed with ethanol (right). Representative results of five patients are shown. (TIF) [file pone.0088548.s001.tif]

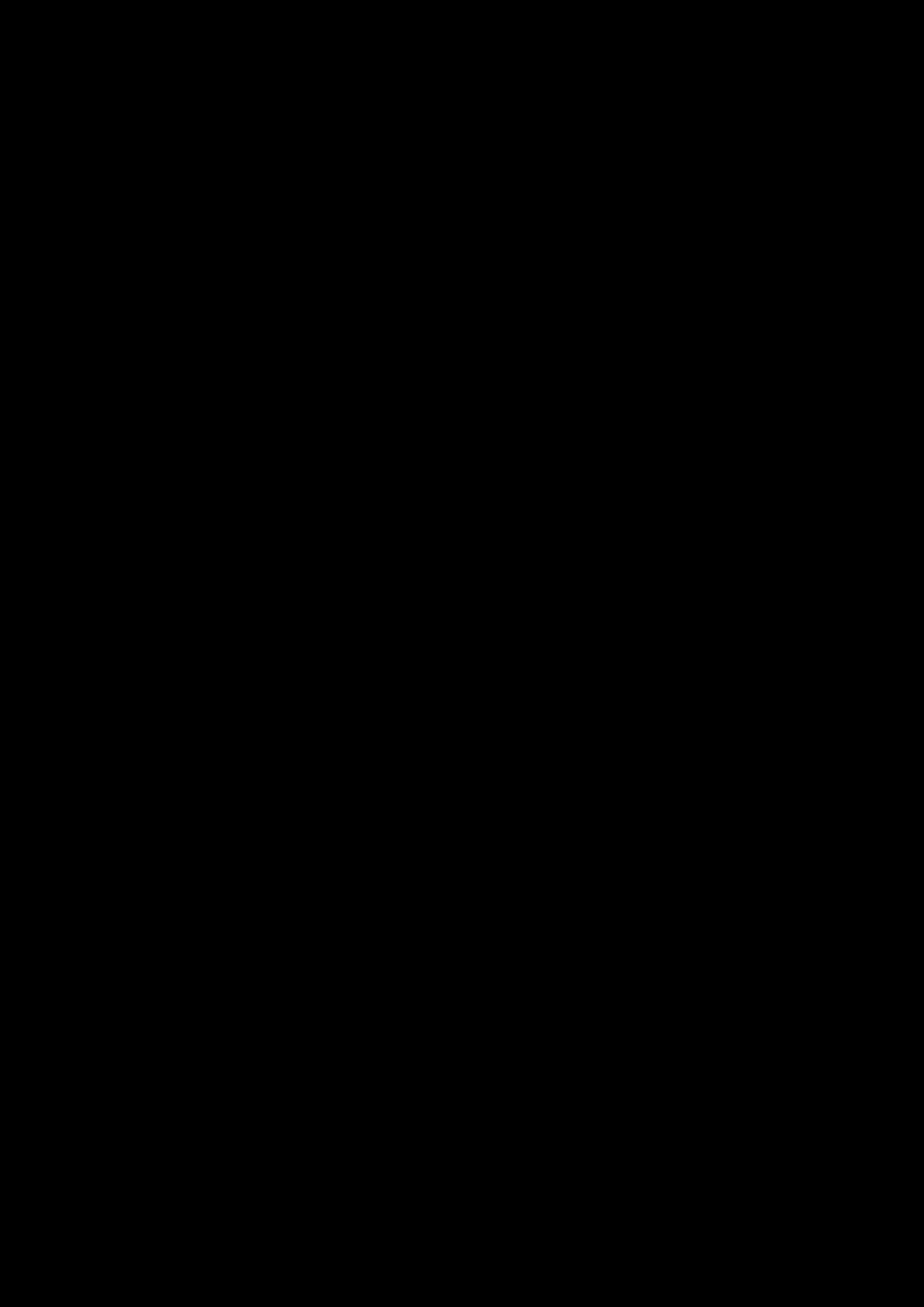

Supplement: Figure S2 — Malaria autoantibodies are of IgG isotype. Malaria related pattern on HEp-2 cells obtained with the whole serum (left) and corresponding isolated IgG (right). Representative results of five patients are shown. (TIF) [file pone.0088548.s002.tif]
