# Supplementary material for: Chronic Malaria Revealed by a New Fluorescence Pattern on the Antinuclear Autoantibodies Test
Source: PLoS One. 2014 Feb 13;9(2):e88548. doi: 10.1371/journal.pone.0088548 (PMC3923801; doi:10.1371/journal.pone.0088548)
Supplement: Table S1 — ANA testings on HEp2000 cells of sera from patients with schistosomiasis, toxocarosis, leishmaniasis, filariasis or leishmaniasis. (DOCX) [file pone.0088548.s003.docx]

Table SI

|  | Nuclear Aspect | | | | | | Cytoplasmic aspect | | | |  |
| --- | --- | --- | --- | --- | --- | --- | --- | --- | --- | --- | --- |
|  | ANA positive (threshold 1/160) | Negative | Homogenous and speckled | Speckled | Nucleolus | Others | Perinuclear enhancement | Diffuse cytoplasmic | Ponctuated cytoplasmic | Other cytoplasmic | Nucleo-Cytoplasmic pattern |
| Toxocariasis N=50 | 32 % (16/50) | 17 | 9 | 16 | 4 | 3 | 0 | 1 | 0 | 7 | 0 |
| Schistosomiasis  N=40 | 20% (8/40) | 16 | 0 | 19 | 1 | 4 | 0 | 0 | 0 | 1 | 0 |
| Filariasis  N=8 | 12.5% (1/8) | 5 | 0 | 3 | 0 | 0 | 0 | 0 | 0 | 2 | 0 |
| Leishmaniasis N=10 | 90% (9/10) | 1 | 0 | 1 | 5 | 3 | 0 | 5 | 0 | 0 | 0 |
| Chagas disease  N=44 | 38.6% (17/44) | 12 | 11 | 19 | 7 | 3 | 0 | 2 | 2 | 1 | 0 |
