# Supplementary material for: Chronic Malaria Revealed by a New Fluorescence Pattern on the Antinuclear Autoantibodies Test
Source: PLoS One. 2014 Feb 13;9(2):e88548. doi: 10.1371/journal.pone.0088548 (PMC3923801; doi:10.1371/journal.pone.0088548)
Supplement: Table S2 — Homology of autoantigens recognized by malalarial autoantibodies with plasmodial antigens using BLAST analysis. (DOCX) [file pone.0088548.s004.docx]

| **Gene description** | **Plasmodial homologous protein** | **location** | **length** | **Score** | **Expect** | **p** | **Identities** | **Positives** |
| --- | --- | --- | --- | --- | --- | --- | --- | --- |
| Bardet-Biedl syndrome 10 protein | **T-complex protein 1 epsilon subunit, putative** | Pf3D7_03_v3:845713-847320(+) | 542 | 97 (39.2 bits) | 7.9e-09 | Sum P(4) = 7.9e-09 | 34/161 (21%) | 68/161 (42%) |
| LAG1 homolog, ceramide synthase 6, mRNA (cDNA clone IMAGE:4396549), complete cds. | **translocation associated membrane protein, putative (TRAM)** | Pf3D7_14_v3:133737-135073(-) | 359 | 242 (90.2 bits) | 8.6e-22 | Sum P(2) = 8.6e-22 | 56/194 (28%) | 108/194 (55%) |
| Abhydrolase domain-containing protein 1 | tRNA m2G10 methyltransferase subunit, putative | Pf3D7_14_v3:269881-270927(+) | 125 | 55 (24.4 bits) | 0.999 | P = 0.999 | 15/53 (28%) | 27/53 (50%) |
| lymphotoxin beta (TNF superfamily, member 3) (LTB), transcript variant 2 | stevor | Pf3D7_01_v3:87203-88177(-) | 289 | 50 (22.7 bits) | 0.37 | P = 0.31 | 13/29 (44%) | 19/29 (65%) |
| RAB11B, member RAS oncogene family (RAB11B) | **Rab GTPase 11b (RAB11b)** | Pf3D7_13_v3:1621292-1622718(-) | 216 | 682 (245.1 bits) | 5.9e-69 | P = 5.9e-69 | 140/217 (64%) | 171/217 (78%) |
| PREDICTED: Homo sapiens hypothetical protein LOC284749 (LOC284749) | conserved Plasmodium protein, unknown function | Pf3D7_11_v3:1275574-1275663(+) | 29 | 35 (17.4 bits) | 0.995 | P = 0.995 | 7/11 (63%) | 9/11 (81%) |
| ALS2 C-terminal like (ALS2CL), transcript variant 3 | **MORN repeat-containing protein 1 (MORN1)** | Pf3D7_10_v3:1257384-1258478(+) | 364 | 252 (93.8 bits) | 8.1e-21 | P = 8.1e-21 | 65/220 (29%) | 106/220 (48%) |
| RNA binding motif protein 33 (RBM33) | **interspersed repeat antigen (FIRA)** | Pf3D7_05_v3:74509-79842(+) | 1720 | 81 (33.6 bits) | 1.1e-08 | Sum P(2) = 1.1e-08 | 123/560 (21%) | 204/560 (36%) |
| magnesium-dependent phosphatase 1 (MDP-1) | P36-like protein homologue, putative | Pf3D7_07_v3:926692-928028(+) | 214 | 68 (29.0 bits) | 0.088 | Sum P(2) = 0.084 | 13/53 (24%) | 28/53 (52%) |
| BTB/POZ domain-containing protein KCTD17 | **kelch protein, putative** | Pf3D7_13_v3:1724817-1726997(-) | 726 | 168 (64.2 bits) | 5.8e-11 | Sum P(2) = 5.8e-11 | 37/87 (42%) | 55/87 (63%) |
| Sperm surface protein Sp17 | **conserved Plasmodium protein, unknown function** | Pf3D7_11_v3:175248-175433(+) | 1381 | 103 (41.3 bits) | 1.2e-05 | P =1.2e-05 | 24/75 (32%) | 43/75 (57%) |
| polymerase (DNA directed), alpha 2 (70kD subunit) (POLA2) | **DNA polymerase alpha subunit, putative** | Pf3D7_14_v3:2572988-2574607(+) | 539 | 204 (76.9 bits) | 5.4e-20 | Sum P(2) = 5.4e-20 | 65/250 (26%) | 123/250 (49%) |
| Zinc finger and SCAN domain-containing protein 2 | **zinc finger transcription factor (KROX1)** | Pf3D7_12_v3:428495-432880(-) | 1461 | 262 (97.3 bits) | 2.6e-20 | Sum P(2) = 2.6e-20 | 92/380 (24%) | 159/380 (41% |
| Heat shock protein HSP 90-alpha | **heat shock protein 90 (HSP90)** | Pf3D7_07_v3:381592-384614(+) | 745 | 1711 (607.4 bits) | 5.4e-178 | Sum P(2) = 5.4e-178 | 329/518 (63%) | 416/518 (80%) |
| coiled-coil-helix-coiled-coil-helix domain containing 4 (CHCHD4) | erythrocyte membrane protein 1, PfEMP1 (VAR) | Pf3D7_04_v3:545987-553810(-) | 2209 | 75 (31.5 bits) | 0.078 | P = 0.078 | 24/92 (26%) | 42/92 (45%) |
| zinc finger protein 167 (ZNF167) | **zinc finger transcription factor (KROX1)** | Pf3D7_12_v3:428495-432880(-) | 1461 | 265 (98.3 bits) | 5.4e-21 | Sum P(2) = 5.4e-21 | 67/228 (29%) | 100/228 (43%) |
| pre-B-cell colony enhancing factor 1 (PBEF1) | conserved Plasmodium protein, unknown function | Pf3D7_14_v3:487776-490380(-) | 760 | 69 (29.3 bits) | 0.027 | Sum P(3) = 0.027 | 28/105 (26%) | 46/105 (43%) |
| ubiquitin-conjugating enzyme E2, J2 (UBC6 homolog, yeast) (UBE2J2), transcript variant 4 | **ubiquitin conjugating enzyme E2, putative (UBC)** | Pf3D7_08_v3:631584-632229(-) | 163 | 185 (70.2 bits) | 2.8e-16 | P = 2.8e-16 | 36/118 (30%) | 68/118 (57%) |
| 39S ribosomal protein L30, mitochondrial | conserved Plasmodium protein, unknown function | Pf3D7_09_v3:977869-979965(-) | 698 | 63 (27.2 bits) | 3.2 | P = 0.96 | 26/96 (27%) | 40/96 (41%) |
| Glucose-fructose oxidoreductase domain-containing protein 1 | conserved Plasmodium protein, unknown function | Pf3D7_12_v3:851012-854739(-) | 711 | 65 (27.9 bits) | 8.0 | P = 0.9997 | 12/39 (30%) | 24/39 (61%) |
| ankyrin repeat domain 50 (ANKRD50) | **ankyrin-repeat protein, putative** | Pf3D7_06_v3:1089190-1094328(+) | 1712 | 258 (95.9 bits) | 3.5e-18 | Sum P(2) = 3.5e-18 | 86/300 (28%) | 146/300 (48%) |
| ADP-ribosylation factor-like 9 (ARL9) | ADP-ribosylation factor, putative | Pf3D7_10_v3:1378759-1379295(+) | 178 | 243 (90.6 bits) | 2.0e-22 | P = 2.0e-22 | 56/151 (37%) | 87/151 (57%) |
| SMYD family member 5 (SMYD5) | **SET domain protein, putative (SET7)** | Pf3D7_13_v3:2195449-2196978(+) | 509 | 127 (49.8 bits) | 9.5e-07 | Sum P(2) = 9.5e-07 | 28/67 (41%) | 36/67 (53%) |
| PREDICTED: Homo sapiens hypothetical LOC387942 (LOC387942) | NR |  |  |  |  |  |  |  |
| palmitoyl-protein thioesterase 2 (PPT2), transcript variant 2 | transcription activator, putative | Pf3D7_13_v3:1677540-1677917(-) | 58 | 44 (20.5 bits) | 0.79 | P = 0.79 | 9/28 (32%) | 15/28 (53%) |
| G1/S-specific cyclin-D1 | conserved Plasmodium protein, unknown function | Pf3D7_14_v3:690451-691272(+) | 273 | 70 (29.7 bits) | 0.38 | P = 0.32 | 24/101 (23%) | 50/101 (49%) |
| PREDICTED: Homo sapiens hypothetical LOC401607 (LOC401607) | NR |  |  |  |  |  |  |  |
| vezatin, adherens junctions transmembrane protein (VEZT) | **conserved Plasmodium protein, unknown function** | Pf3D7_09_v3:168493-172887(+) | 742 | 135 (52.6 bits) | 2.0e-06 | P = 2.0e-06 | 67/302 (22%) | 142/302 (47%) |
| cAMP responsive element modulator (CREM), transcript variant 22 | product=liver stage antigen 3 (LSA3) | Pf3D7_02_v3:796752-801586(+) | 1558 | 86 (35.3 bits) | 0.029 | P = 0.029 | 58/264 (21%) | 106/264 (40%) |
| ATP synthase, H+ transporting, mitochondrial F0 complex, subunit d (ATP5H), nuclear gene encoding mitochondrial protein, transcript variant 1 | DNA helicase, putative | Pf3D7_06_v3:198862-204355(+) | 1712 | 92 (37.4 bits) | 0.0034 | P=0.0034 | 34/112 (30%) | 61/112 (54%) |
| KIAA1598 (KIAA1598) | **conserved Plasmodium protein, unknown function** | Pf3D7_05_v3:180421-187470(+) | 2349 | 248 (92.4 bits) | 1.0e-17 | P = 1.0e-17 | 92/354 (25%) | 190/354 (53%) |
| STE20-like kinase (yeast) (SLK) | **calcium dependent protein kinase 3 (CDPK3)** | Pf3D7_03_v3:422379-424680(+) | 562 | 385 (140.6 bits) | 1.1e-34 | P = 1.1e-34 | 91/268 (33%) | 142/268 (52%) |
| histone deacetylase 4 (HDAC4) | **histone deacetylase, putative** | Pf3D7_10_v3:321011-328150(+) | 2379 | 249 (92.7 bits) | 1.6e-39 | Sum P(4) = 1.6e-39 | 40/78 (51%) | 54/78 (69%) |
| hypothetical protein LOC205251 (LOC205251) | NR |  |  |  |  |  |  |  |
| PREDICTED: Homo sapiens hypothetical LOC285307 (LOC285307) | NR |  |  |  |  |  |  |  |
| peroxiredoxin 2 (PRDX2) | **thioredoxin peroxidase 1 (Trx-Px1)** | Pf3D7_14_v3:1575984-1576571(+) | 195 | 484 (175.4 bits) | 5.7e-48 | P = 5.7e-48 | 91/191 (47%) | 131/191 (68%) |
| eukaryotic translation initiation factor 4 gamma, 3 (EIF4G3) | **polyadenylate-binding protein-interacting protein 1, putative (PAIP1)** | Pf3D7_11_v3:301283-311287(-) | 3334 | 297 (109.6 bits) | 2.3e-21 | Sum P(3) = 2.3e-21 | 94/299 (31%) | 154/299 (51%) |
| PREDICTED: Homo sapiens similar to CG14853-PB (LOC285141) | **product=MSP7-like protein** | Pf3D7_13_v3:1400897-1402276(-) | 459 | 118 (46.6 bits) | 3.1e-08 | P=3.1e-08 | 38/144 (26%) | 62/144 (43%) |
| Interferon-induced guanylate-binding protein 2 | **conserved Plasmodium protein, unknown function** | Pf3D7_11_v3:756192-760435(+) | 1070 | 156 (60.0 bits) | 3.2e-09 | Sum P(4) = 3.2e-09 | 55/235 (23%) | 124/235 (52%) |
| Uncharacterized protein C14orf104 | **conserved Plasmodium protein, unknown function** | Pf3D7_09_v3:1270155-1272656(+) | 715 | 125 (49.1 bits) | 1.5e-07 | Sum P(3) =1.5e-07 | 51/197 (25%) | 97/197 (49%) |
| MRG-binding protein | Plasmodium protein, unknown function | Pf3D7_12_v3:1871919-1872911(+) | 222 | 78 (32.5 bits) | 0.020 | P = 0.020 | 33/135 (24%) | 57/135 (42%) |
| Olfactory receptor 8D1 | guanylyl cyclase beta (GCbeta) | Pf3D7_13_v3:2413324-2425261(+) | 3179 | 72 (30.4 bits) | 0.073 | Sum P(2) = 0.070 | 35/137 (25%) | 58/137 (42%) |
